# Endovascular Aortic Repair under Extracorporeal Cardiac Support in a Patient with an Abdominal Aortic Aneurysm Impending Rupture and Aortic Stenosis: A Case Report

**DOI:** 10.3400/avd.cr.20-00059

**Published:** 2020-09-25

**Authors:** Yohei Kawatani, Motoshige Yamasaki, Atsushi Oguri

**Affiliations:** 1 Department of Cardiovascular Surgery, Takasaki Heart Hospital; 2 Department of Cardiology, Takasaki Heart Hospital

**Keywords:** endovascular abdominal aortic aneurysm repair, EVAR, ECMO

## Abstract

Aortic stenosis is a serious valvular disease that increases the risk of cardiac arrest and/or cardiogenic shock during noncardiac surgery. A 93-year-old woman with an abdominal aortic aneurysm impending rupture and aortic stenosis underwent endovascular abdominal aortic aneurysm repair. During surgery, the patient presented with ventricular tachycardia. Due to on-going cardiogenic shock, we did a direct cannulation into the right axillary artery for the immediate establishment of venoarterial extracorporeal membrane oxygenation. The endovascular treatment of the abdominal aortic aneurysm was completed according to the standard procedure. The patient recovered without any complications, including heart failure or neurological dysfunction.

## Introduction

In patients with aortic stenosis (AS), noncardiac surgery poses a high risk.^1,2)^ In those suffering simultaneously from AS and abdominal aortic aneurysm (AAA), the AAA should be urgently repaired. However, such patients undergoing operation are at high risk owing to the likelihood of arrhythmia, cardiogenic shock, or cardiac arrest. Here, we report a successful case of endovascular aortic repair (EVAR) of an AAA performed under extracorporeal cardiac support due to cardiac arrest and cardiogenic shock during the surgery.

## Case Report

**Figure 1:**
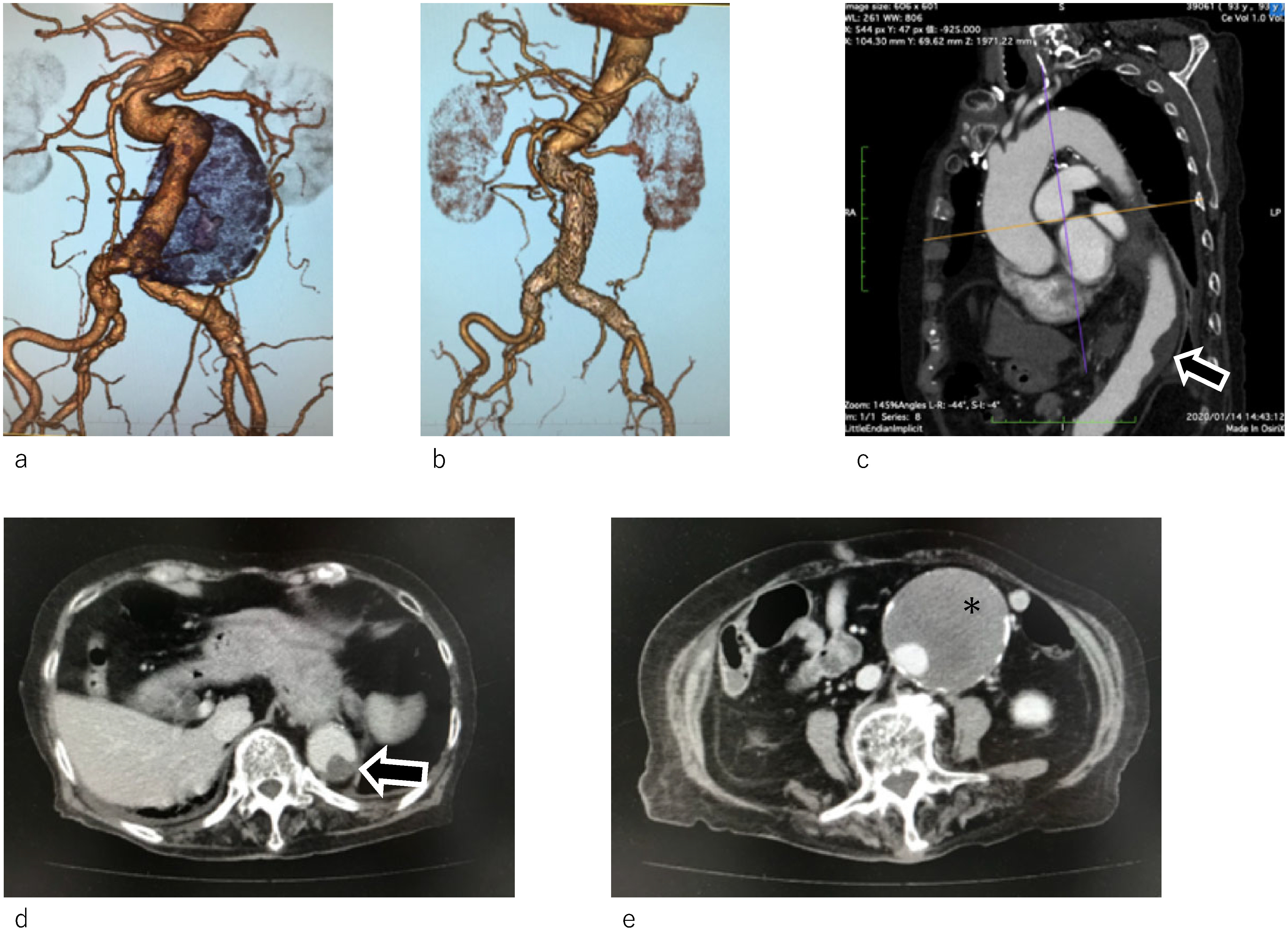
Fig. 1 Pre- and postoperative computed tomography (CT) images: (**a, b**) Preoperative and postoperative enhanced CT three dimensional images. We successfully placed the stent-grafts as planned. (**c**–**e**) Preoperative CT images for analysis of the aorta. We observed a large thrombus in the descending aorta (arrow) and in the aneurysm(*).

A 93-year-old woman with severe back pain was diagnosed as AAA with an impending rupture (**Figs. 1a and 1b**). Ultrasound cardiography revealed moderate AS. The detailed results of the examination are as follows: valve area: 1.56 cm^2^; peak velocity: 3.05 m/s; peak pressure gradient: 37 mmHg; left ventricular end-diastolic diameter: 42 mm; and left ventricular end-systolic diameter: 26 mm; normal wall motion was seen. The patient had peripheral arterial disease in the lower limb; the right/left ankle brachial blood pressure index was 0.67/0.85, respectively. A closer evaluation of the enhanced computed tomography (CT) images revealed a massive thrombus in the descending aorta and in the AAA (**Figs. 1c–1e**).

We performed EVAR, and began the surgery with an immediate stand-by of extracorporeal cardiac support if necessary. The surgery was performed under general anesthesia. The patient was sterilized from the shoulder to the upper thigh, which enabled us to expose the axillary artery for inflow cannulation during the surgery. The right femoral vein was also prepared for outflow cannulation during exposure of the right femoral artery for access rout for EVAR.

**Figure 2:**
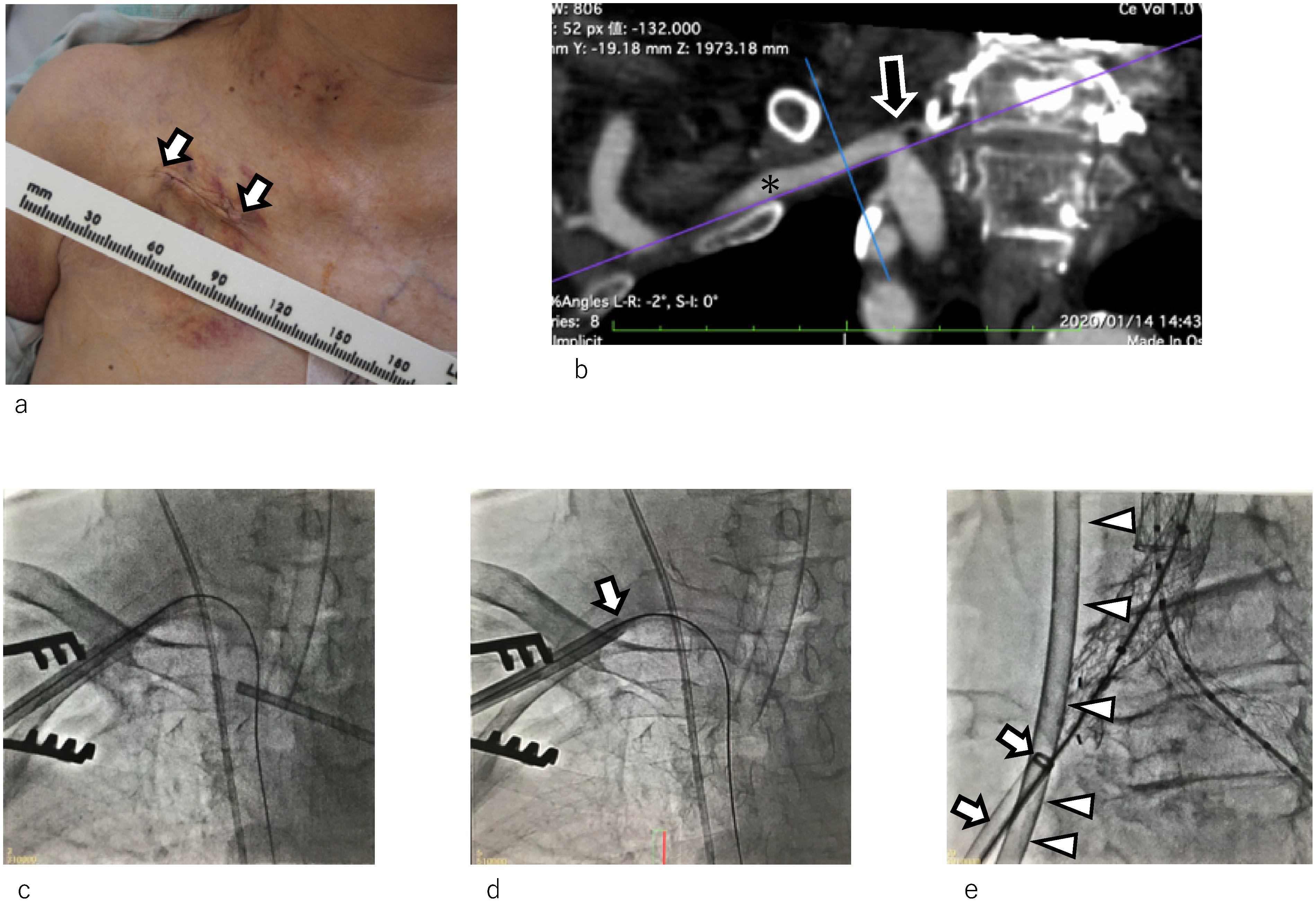
Fig. 2 Images during the surgery: (**a**) An image of the wound for the right axillary artery exposure and cannulation (arrows). (**b**) Preoperative computed tomography (CT) images of the right axillary and subclavian artery. An asterisk (*) denotes the planned cannulation site, and an arrow denotes the vertebral artery. (**c**) Fluoroscopy image during insertion of the inflow cannula. The cannula was inserted using the direct Seldinger method. (**d**) Fluoroscopy image after placement of the inflow cannula. (**e**) Fluoroscopy image during endovascular stent-graft placement. Arrow heads: 21 Fr outflow cannula, arrows: 16 Fr guiding sheath for stent-graft placement.

Eight Fr sheaths (Terumo, Tokyo, Japan) were inserted into both femoral arteries. A stiff wire (Boston Scientific, Marlborough, MA, USA) was inserted from the right side, and a pigtail catheter was inserted from the left side. An arteriogram was performed. At this point, the patient presented with ventricular tachycardia (VT), and the systolic blood pressure dropped from 100 to 30 mmHg (arterial line pressure monitor). The VT recovered to sinus rhythm spontaneously, but cardiac shock was prolonged. We decided to initiate extracorporeal cardiac support. A 4-cm-long skin incision was made in the precordium beneath and along the right clavicle (**Fig. 2a**). Through the incision, we exposed the right axillary artery, which was 6×7 mm in diameter on the preoperative enhanced CT (**Fig. 2b**). Under X-ray fluoroscopy, an inflow cannula (Capiox 16.5 Fr×15 cm; Terumo) was inserted using the direct Seldinger technique (**Fig. 2c**). The tip was placed carefully at the distal portion of the subclavian artery, which was assumed distal enough from the right carotid and the right vertebral artery according to preoperative CT (**Figs. 2b–2d**). The outflow cannula (Capiox 21 Fr×50 cm; Terumo) was inserted into the right common femoral vein (**Fig. 2e**), and extracorporeal circulation was initiated. The length of time between deciding to initiate the extracorporeal cardiac support and the flow reaching the adequate level was 12 min. Thereafter, the blood pressure was maintained between 80 and 140 mmHg.

The EVAR procedure was successfully completed. Cannulas did not disturb the procedure. A GORE EXCLUDER AAA endoprosthesis (W. L. Gore & Associates, Flagstaff, AZ, USA) was used, 24 mm for proximal and 16 mm for both distal landings. All procedures were performed carefully to prevent any device from contacting the thrombus in the descending aorta. After we confirmed the stabilization of the hemodynamic status, extracorporeal cardiac support was terminated. The operation time was 175 min, and the extracorporeal circulation time was 72 min.

The patient recovered from the operation without any complications and did not experience subsequent VT or heart failure. Moreover, the back pain also subsided, and an enhanced CT showed successful treatment of the abdominal aortic aneurysm (**Fig. 1b**). The patient did not experience any major adverse cardiovascular event at the 45-day follow-up.

## Discussion

In patients with heart disease, patients undergoing aortic surgery and peripheral artery surgery are categorized into the high-risk-surgery group.^3–5)^ On the other hand, AS poses a risk for cardiogenic shock or cardiac arrest during the operation. Moreover, moderate AS is associated with poorer primary outcomes, defined as the composite of 30-day mortality and postoperative myocardial infarction, compared with no AS (4.4% versus 1.7%; P=0.002).^6)^ According to the guidelines for perioperative cardiovascular evaluation and management of noncardiac surgery published by the Japanese circulation society, patients with moderate AS can undergo noncardiac surgery owing to the invasiveness and urgency of the surgery.^5)^ Thus, we performed EVAR with back up extracorporeal cardiac support that could be started immediately, if necessary. The strategy improved the hemodynamics, and EVAR was performed successfully. Abouliatim et al. reported a case where open AAA repair under venoarterial extracorporeal membrane oxygenation (V-A ECMO) was performed after a failed EVAR attempt.^6)^ The authors used V-A ECMO with right axillary artery perfusion and inferior vena cava drainage through the right femoral vein. To our knowledge, our case is the first report of successful EVAR under extracorporeal cardiac support.

During the surgery, the patient experienced VT and a blood pressure drop, resulting in cardiac arrest, and cardiac shock was prolonged. We assumed that this was caused by changes in factors affecting the hemodynamic state as follows: general anesthesia, invasiveness of the operation, and manipulation of femoral arteries. Additionally, after initiating the EVAR, we should have inserted a larger balloon and larger sheaths and inflated the larger balloon in the aorta. We assumed that continuing the EVAR procedure was causing the severe changes in the hemodynamics. Therefore, we decided to activate the extracorporeal cardiac support.

For cardiac support, we chose V-A ECMO. The method is partial cardio-pulmonay bypass commonly used in the minimal invasive cardiac surgery or surgery for thoracoabdominal aoritic aneurysm (F–F bypass), and can adequately support circulation and oxygenation even under cardiac arrest. At the same time, it can reduce cardiac preload by draining blood from the superior or inferior vena cava; however, the technique can increase the left ventricular afterload,^6,7)^ and should, therefore, be performed carefully. As there are no standard recommendations regarding blood pressure levels in such a situation, we set the lower limit at 80 mmHg and the upper limit at 140 mmHg, considering the patient’s preoperative blood pressure as a guide. Arterial O_2_ saturation was closely monitored. After the hemodynamic status (flow rate at cardiac index 1.5–1.8 L/min/m^2^) had stabilized, the blood pressure gradually increased. We controlled the extracorporeal circulation flow carefully so as not to exceed 140 mmHg. To further reduce the cardiac overload, nicardipine was used.

We selected the right axillary artery for perfusion and cannulated it using the direct Seldinger technique. This technique was useful in maintaining V-A ECMO and continue the EVAR procedure and prevent complications associated with peripheral artery perfusion. The femoral artery is commonly used in V-A ECMO, especially in emergency settings,^8)^ but the perfusion causes retrograde blood flow into the aorta, which is associated with a risk of cerebral stroke.^9)^ In addition, femoral artery cannulation can cause limb ischemia.^10,11)^ In such a case, the axillary artery is a good inflow site, because perfusion into this artery causes antegrade blood flow into the aortic arch.^12,13)^ Moreover, using the axillary artery for perfusion, the femoral arteries were able to be used as access routes for the EVAR.

Although our approach was successful, transesophageal echocardiogram could have been useful. Abouliatim et al. reported that in their open repair of an AAA under V-A ECMO support, transesophageal echocardiogram was useful to monitor the status of the heart.^6)^ We had planned to use the Swan–Ganz catheter only if weaning from the V-A ECMO was difficult or if the hemodynamics were not stable. In fact, we did not use the Swan–Ganz catheter in our case because the hemodynamics status was stabilized after initiation of V-A ECMO. These methods are useful in patients with a high risk of cardiac shock during the surgery in monitoring the hemodynamic status to reduce the risk of deterioration of the cardiac status and to monitor the hemodynamic status after the initiation of cardiac support devices.

## Conclusion

In a patient who experienced cardiac arrest and cardiogenic shock during EVAR, we successfully completed the surgery under partial cardiopulmonary bypass with femoral venous drainage and axillary arterial perfusion.

## Data Availability

All the data supporting our conclusions are presented in the article.

## References

[1] Torsher LC, Shub C, Rettke SR, et al. Risk of patients with severe aortic stenosis undergoing noncardiac surgery. Am J Cardiol 1998; 81: 448-52. 9485135 10.1016/s0002-9149(97)00926-0

[2] Rohde LE, Polanczyk CA, Goldman L, et al. Usefulness of transthoracic echocardiography as a tool for risk stratification of patients undergoing major noncardiac surgery. Am J Cardiol 2001; 87: 505-9. 11230829 10.1016/s0002-9149(00)01421-1

[3] Fleisher LA, Beckman JA, Brown KA, et al. ACC/AHA 2007 guidelines on perioperative cardiovascular evaluation and care for noncardiac surgery: a report of the American College of Cardiology/American Heart Association Task Force on Practice Guidelines (Writing Committee to Revise the 2002 Guidelines on Perioperative Cardiovascular Evaluation for Noncardiac Surgery): developed in collaboration with the American Society of Echocardiography, American Society of Nuclear Cardiology, Heart Rhythm Society, Society of Cardiovascular Anesthesiologists, Society for Cardiovascular Angiography and Interventions, Society for Vascular Medicine and Biology, and Society for Vascular Surgery. Circulation 2007; 116: e418-99. 17901357 10.1161/CIRCULATIONAHA.107.185699

[4] Poldermans D, Bax JJ, Boersma E, et al. Guidelines for pre-operative cardiac risk assessment and perioperative cardiac management in non-cardiac surgery. Eur Heart J 2009; 30: 2769-812. 19713421 10.1093/eurheartj/ehp337

[5] Kyo S, Imanaka K, Masuda M, et al. Guidelines for Perioperative Cardiovascular Evaluation and Management for Noncardiac Surgery (JCS 2014). Circ J 2017; 81: 245-67. 28111417 10.1253/circj.CJ-66-0135

[6] Abouliatim I, Paramythiotis A, Harmouche M, et al. Extracorporeal membrane oxygenation support for abdominal aortic aneurysms surgery in high-risk patients. Interact Cardiovasc Thorac Surg 2012; 14: 215-6. 22159237 10.1093/icvts/ivr031 PMC3279988

[7] Fuhrman BP, Hernan LJ, Rotta AT, et al. Pathophysiology of cardiac extracorporeal membrane oxygenation. Artif Organs 1999; 23: 966-9. 10564298 10.1046/j.1525-1594.1999.06484.x

[8] Pavlushkov E, Berman M, Valchanov K. Cannulation techniques for extracorporeal life support. Ann Transl Med 2017; 5: 70. 28275615 10.21037/atm.2016.11.47 PMC5337209

[9] Murzi M, Cerillo AG, Miceli A, et al. Antegrade and retrograde arterial perfusion strategy in minimally invasive mitral-valve surgery: a propensity score analysis on 1280 patients. Eur J Cardiothorac Surg 2013; 43: e167-72. 23404687 10.1093/ejcts/ezt043

[10] Gander JW, Fisher JC, Reichstein AR, et al. Limb ischemia after common femoral artery cannulation for venoarterial extracorporeal membrane oxygenation: an unresolved problem. J Pediatr Surg 2010; 45: 2136-40. 21034934 10.1016/j.jpedsurg.2010.07.005 PMC4297677

[11] Bisdas T, Beutel G, Warnecke G, et al. Vascular complications in patients undergoing femoral cannulation for extracorporeal membrane oxygenation support. Ann Thorac Surg 2011; 92: 626-31. 21550582 10.1016/j.athoracsur.2011.02.018

[12] Sabik JF, Lytle BW, McCarthy PM, et al. Axillary artery: an alternative site of arterial cannulation for patients with extensive aortic and peripheral vascular disease. J Thorac Cardiovasc Surg 1995; 109: 885-90; discussion, 890-1. 7739248 10.1016/S0022-5223(95)70312-8

[13] Ramchandani M, Al Jabbari O, Abu Saleh WK, et al. Cannulation strategies and pitfalls in minimally invasive cardiac surgery. Methodist DeBakey Cardiovasc J 2016; 12: 10-3. 10.14797/mdcj-12-1-10 PMC4847960 27127556

